# Naming ability assessment in neurocognitive disorders: a clinician’s perspective

**DOI:** 10.1186/s12888-022-04486-x

**Published:** 2022-12-30

**Authors:** Eliza ( Eleni-Zacharoula) Georgiou, Savvina Prapiadou, Vasileios Thomopoulos, Maria Skondra, Marina Charalampopoulou, Asimina Pachi, Αlexandra Anagnostopoulou, Theofanis Vorvolakos, Robert Perneczky, Antonios Politis, Panagiotis Alexopoulos

**Affiliations:** 1 Department of Psychiatry, Patras University General Hospital, Faculty of Medicine, School of Health Sciences, University of Patras, Patras, Greece; 2 Large-Scale Machine Learning & Cloud Data Engineering Laboratory (ML@Cloud-Lab), Faculty of Computer Engineering & Informatics, School of Engineering, University of Patras, Patras, Greece; 3 General Hospital of Zakynthos “Saint Dionysios”, Zakynthos, Greece; 4 Department of Psychiatry, Faculty of Medicine, School of Health Sciences, University Hospital of Alexandroupolis, Democritus University of Thrace, Alexandroupolis, Greece; 5 Division of Mental Health in Older Adults and Alzheimer Therapy and Research Center, Department of Psychiatry and Psychotherapy, University Hospital, Ludwig-Maximilians-Universität Munich, Munich, Germany; 6 Ageing Epidemiology (AGE) Research Unit, School of Public Health, Faculty of Medicine, The Imperial College of Science, Technology and Medicine, London, UK; 7 German Center for Neurodegenerative Diseases (DZNE) Munich, Munich, Germany; 8 Munich Cluster for Systems Neurology (SyNergy), Munich, Germany; 9 Sheffield Institute for Translational Neurosciences (SITraN), University of Sheffield, Sheffield, UK; 10 First Department of Psychiatry, Eginition Hospital, School of Medicine, National and Kapodistrian University of Athens, Athens, Greece; 11 Department of Psychiatry, Division of Geriatric Psychiatry and Neuropsychiatry, Johns Hopkins Medical School, Baltimore, USA; 12 Global Brain Health Institute, Medical School, Trinity College Dublin, The University of Dublin, Dublin, Republic of Ireland; 13 Department of Psychiatry and Psychotherapy, Klinikum rechts der Isar, Faculty of Medicine, Technical University of Munich, Munich, Germany; 14 Patras Dementia Day Care Center, Corporation for Succor and Care of Elderly and Disabled – FRODIZO, Patras, Greece

**Keywords:** dysnomia, Word-finding, Dementia, Major and mild neurocognitive disorder, anomia

## Abstract

**Background:**

Detecting impaired naming capacity is valuable in diagnosing neurocognitive disorders (ND). A.

clinical practice- oriented overview of naming tests validated in ND is not available yet. Here, features of naming tests with validated utility in ND which are open access or available for purchase are succinctly presented and compared.

**Methods:**

Searches were carried out across Pubmed, Medline and Google Scholar. Additional studies were identified by searching reference lists. Only peer-reviewed journal articles were eligible. A narrative- and tabullar synthesis was used to summarize different aspects of the naming assessment instruments used in patients with ND such as stimuli type, administration time,

assessment parameters and accessibility. Based on computational word frequency calculations, the tests were compared in terms of the average frequency of their linguistic content.

**Results:**

Twelve naming tests, relying either on visual or auditory stimuli have been validated in ND. Their content and administration time vary between three and 60 items and one and 20 minutes, respectively. The average frequency of the words of each considered test was two or lower, pointing to low frequency of most items. In all but one test, scoring systems are exclusively based on correctly named items. Seven instruments are open access and four are available in more than one language.

**Conclusions:**

Gaining insights into naming tests’ characteristics may catalyze the wide incorporation of those with short administration time but high diagnostic accuracy into the diagnostic workup of ND at primary healthcare and of extensive, visual or auditory ones into the diagnostic endeavors of memory clinics, as well as of secondary and tertiary brain healthcare settings.

**Supplementary Information:**

The online version contains supplementary material available at 10.1186/s12888-022-04486-x.

## Background

The assessment of naming capacity is important for diagnosing neurocognitive disorders (ND) and may contribute to identifying their cause. Even though a mild decline in word-finding capacity is related to aging [1, 2], more severe naming deficits embody core symptoms of a number of ND. For instance, in Alzheimer’s disease (AD) [3, 4] dysnomia is a symptom that appears early in the symptomatic stages of the disease as indicated by the reported significant dysnomia in patients with mild neurocognitive disorder (MiND) due to AD [5–7]. In addition, dysnomia is the leading symptom in the semantic and logopenic variants of primary progressive aphasia (PPA), the language variant of frontotemporal lobar degeneration (FTLD) [8], and is frequently observed even in the behavioral FTLD form [9]. Moreover, naming deficits can precede full blown major neurocognitive disorder (MaND) due to Lewy bodies [10, 11] and Parkinson’s disease [12, 13] and can belong to the clinical manifestations of vascular lesions in the language-dominant hemisphere [14]. Furthermore, mild traumatic brain injury and further acquired brain injuries may also lead to dysnomia [15, 16]. In contrast, the subcortical vascular dementia and Huntington’s disease do not typically include naming deficits. Hence, assessment of naming capacity is an important part of the ND diagnostic workup and a valuable tool in detecting their cause. In line with its presence in different clinical entities, dysnomia does not pertain either to pathological alterations in a single brain region or to a one-size-fits-all pathomechanism.

**Fig. 1:**
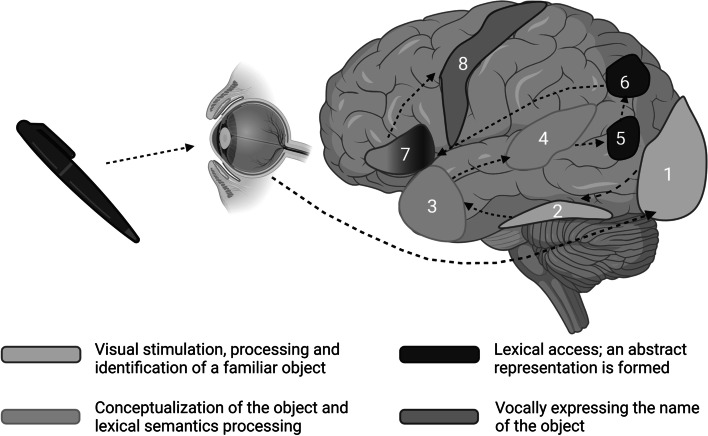
The neuroanatomical pathways involved in the stages of recognizing, processing and executing the output of a stimulus in the multifaceted naming process. The figure was created with BioRender.com

Naming of objects is a multifaceted process which has been deciphered through various imaging techniques (e.g. magnetic resonance imaging, positron emission tomography, electroencephalogram) [16]. This complex task starts with the recognition and perception of an object and ends with articulating the words that describe it (Fig. 1 )[17]. Visual recognition of an object is dependent on visual cortex activation, while occipitotemporal/ fusiform regions are involved in object perception and recognition as something familiar. For instance, neurodegeneration with Lewy bodies that affects the occipitotemporal/ fusiform regions disrupts the visuoperceptual path, leading to inability to name an object [18]. The next step is semantic processing of the object, a path that involves both anterior temporal cortex and posterior superior temporal gyrus. Interestingly, past reports indicate that picture naming is more closely linked to temporoparietal brain regions, while anterior temporal regions may be more closely associated with naming capacity in response to auditory stimuli [19, 20]. Impaired semantic processing leads to semantic errors and paraphasias with relatively intact speech fluency. This phenotype is typically observed in individuals with the semantic variant of PPA [8]. The next step is lexical access in which an abstract representation of the object is formed. This process relies on posterior temporal gyrus, angular gyrus, and inferior frontal gyrus. Dysfunction of this path results in the tip-of-the-tongue phenomenon or the subjective experience of feeling certain that one knows the word but is unable to retrieve it and vocally express it. The tip-of-the-tongue phenomenon is observed in various clinical entities including stroke, MaND due to AD, logopenic PPA and temporal lobe epilepsy. Finally, the last step is to execute the output of the object’s name which involves mainly the posterior inferior frontal cortex. Lesions in this region of the brain result in distorted, agrammatic speech [21].

Several instruments with different characteristics have been designed to assess word-finding capacity. They embody an integral component of the neuropsychological workup of ND [22]. An overview of open access or available to purchase naming tests, the utility of which has been studied in older adults with ND, is presented here. Its novelty hinges in that it directly compares different aspects of the instruments such as stimuli type, administration time and frequency of the words included. The last is based on computational approaches and pertains to the relative difficulty of the words to be named, since word frequency is positively associated with performance on word-finding tests [23, 24]. Words with a high frequency are perceived and generated faster and more efficiently than words with a low frequency [25], and they are conserved in patients’ vocabulary longer than words that are rarely used [23]. The present narrative review was designed and conducted to assist healthcare professionals in identifying the naming test that most properly meets their needs in the ND diagnostic workup in each clinical setting.

## Methods

### Eligibility criteria

The methods of this review followed the Preferred Reporting Items for Systematic Reviews and Meta-analyses (PRISMA) checklist [26]. Eligible studies were studies which focused on stand-alone naming tests or dysnomia tests embedded in global cognitive assessment instruments and which assessed the utility of the dysnomia test in patients with MaND or MiND according to the respective international diagnostic criteria [27–34]. Only peer-reviewed studies on tests which were either open access or available for purchase were considered. Studies which did not explicitly include patients with MaND and/or MiND were excluded from this review (e.g. studies on patients with epilepsy or focal brain lesions but neither MaND nor MiND). In addition, previous reports on global cognitive assessment instruments including dysnomia tests, i.e. screening tools like the Mini-Mental State Examination or test batteries like the Alzheimer’s Disease Assessment Scale–Cognitive Subscale, which had not separately considered the utility of the naming tests embedded in the global cognitive assessment instruments, were not taken into account.

### Search strategy and study selection

Searches were conducted in google scholar, pubmed and medline databases. A focused search strategy was conducted. It was based on the key terms “dysnomia”, “anomia”, “naming” and “MaND”, “MiND”, “dementia”, “mild cognitive impairment”, “cognitive impairment no dementia (CIND)”, “AD”, “vascular cognitive impairment (VCI)”, “FTLD”, “frontotemporal dementia (FTD)”, “PPA”, “semantic dementia”. In addition, the reference lists of eligible studies and systematic reviews related to the key terms were hand searched. Two reviewers (EG, SP) independently screened the title and abstract of potentially eligible studies against the eligibility criteria followed by full-text screening if required. Disagreement between the reviewers was resolved by consulting a third reviewer (PA).

### Data collection process and data items

Data on study design and findings were extracted into a spreadsheet by one reviewer (EG) and checked by a second reviewer (SP). Disagreements were resolved by thorough discussion with a third reviewer (PA). Extracted information included (i) record details (author, title, publication date, journal), (ii) study characteristics (characteristics of the studied instrument [e.g. language, duration, stimuli type], sample size, included diagnostic groups, employed statistical methods), (iii) study findings (diagnostic validity of the studied instrument, [e.g. sensitivity, specificity, detected shortcomings]). Test administration time shorter than 15 minutes was considered to be excellent with regard to respondent burden; satisfactory, if it ranges between 15 and 45 minutes, while lengthy if it lasts longer than 45 minutes [35].

### Word frequency assessment

Word frequency assessment relied on corpora of television subtitles, which is one of the best word frequency measures [36]. Based on American subtitles, Brysbaert & New constructed a frequency measure consisting of 51 million words in total. SUBTLEX_WF_ is the frequency per million words (Subtitle frequency: word form frequency) [37]. The following metrics were used:

- FREQcount. This is the number of times the word appears in the corpus.
- Lg10WF. This value is based on log10(FREQcount+ 1) and has four-digit precision.

Similar databases were used based on German, French and Spanish subtitles for movies and the same metrics for the German [38] French [39] and Spanish language [40, 41]. For some instances of 2-g (and one of a 3-g) the probability of appearance was retrieved from Google Ngram Viewer [42]. The word frequency databases and the files containing the naming tests were imported and processed using Python and the pandas library. The frequency of each item of the naming tests was then checked thoroughly against the respective database and was classified into one of the following frequency categories: 1: rare, 2: infrequent, 3: frequent, 4: very frequent. For the classification of naming test items into these arbitrary chosen four frequency categories, the words of each database were ordered by frequency count and then the first 25% were determined to have rare frequency, the next up to 50% to be infrequent etc. The frequency of ten words is presented in the following lines as an example:

**Table:** 

**Word**	**FREQcount**	**Lg10WF**	**quantile**
accordion	67	1,832,508,913	1
anchor	378	257,863,921	2
ant	273	2,437,750,563	2
apple	1207	3,082,066,934	3
arm	3336	3,523,356,207	4

For each test a total score was calculated based on the ratio of the sum of the frequency of all test items divided by the total number of test items.

## Results

### Study selection

**Fig. 2:**
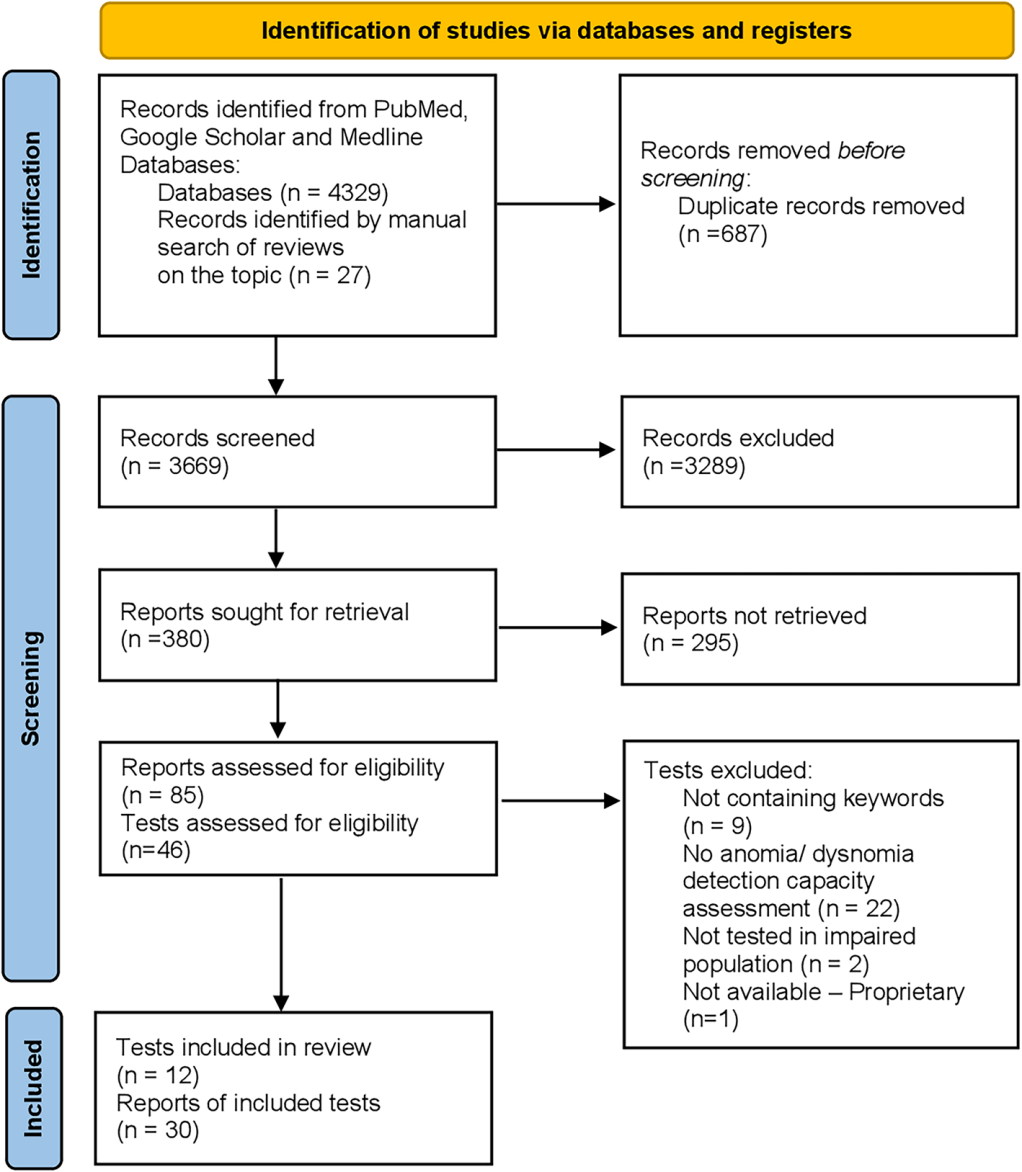
Flow diagram of database and supplementary search and screening results

The database and supplementary searches yielded 72 articles that discussed 46 naming tests (Fig. 2). After the screening of the titles and abstracts of all studies and the full review of these potentially relevant studies, 17 studies and 12 tests fulfilled all eligibility criteria and were included in the review. Of note, three eligible tests mainly the Graded Naming-, Faces- and Buildings Tests were validated together as a single instrument [43]. The main reasons for studies not fulfilling the eligibility criteria were as follows: Two studies were restricted to cognitively healthy individuals, nine did not include patients with MiND or MaND, 22 focused on test batteries including subtests assessing naming capacity but did not report findings concerning the utility of the naming subtest in detecting dysnomia and one was neither open access nor available for purchase (see Supplementary Table 1).

### Characteristics of naming tests and their utility in detecting naming deficits in neurocognitive disorders

The characteristics of naming tests previously used to assess dysnomia in older adults with ND and their diagnostic utility are presented in Table 1. The following tests met the eligibility criteria of the study. Ten were stand-alone dysnomia tests, while two were parts of global cognitive assessment instruments.

**Table 1:** Naming test characteristics and utility in detecting naming deficits in neurocognitive disorders

	Stand-alone dysnomia tests								Dysnomia tests embedded in global cognitive examination tools	
	BNT	ANT	Wo-Fi	MINT 32 items	GBT/ GFT/ GNT	NAB Naming subtest	TDQ-30	VNT	ACE-III Naming subtest	MoCA Naming subtest
**Items**	60	50	50	32	30	31	30	55	16	3
**Stimuli type**	visual	auditory	auditory	visual	visual	visual	visual	visual	visual	visual
**Time (in minutes)**	< 20	< 20	< 15	< 15	< 15	< 10	< 10	< 10	< 5	< 1
**Average test item frequency**	1.717	2.26	1.52	2	2	–	1.75	1.872	2	2
**Assessment parameters**	Correctly named items	Correctly named items, tip-of-the tongue extended latency score, reaction time per response	Correctly named items	Correctly named items	Correctly named items	Correctly named items	Correctly named items	Correctly named items	Correctly named items	Correctly named items
**Access**	Available for purchase Link	Open Link	Open Link	Open Link	Available for purchase GNT Link GFT Link GBT Link	Available for purchase Link	Open Link	Open Link	Open Link	Open Link
**Language Availability**	More languages	English	German	English, Spanish	English	English	French	English	More languages	More languages
**Validation studies**	Knesevich JWm et al. 1986 Erdodi La et al. 2018	Hirsch J et al. 2016 Hirsch J et al. 2021	Camerer-Waldecker C et al. 2019	Stasenko A et al. 2019	Ahmed S et al. 2008	Sachs B et al. 2016 Durant J et al. 2021	MacOir J et al. 2021	Yochim BP et al. 2015 Modore MR et al. 2020	Calderon C et al. 2021	Moafmashhadi P et al. 2013
**Neurocognitive disorders of validation samples**	MiND, MaND due to AD, due to vascular brain disease, due to PD, due to Lewy bodies, due to mixed aetiologies	MiND, MaND due to AD, due to vascular brain disease, due to mixed aetiologies	MaND due to AD	MiND, MaND due to AD	MiND	MiND, MaND due to AD, due to PD, due to Lewy bodies	MiND, MaND due to AD	MiND, MaND due to AD, due to vascular brain disease, due to PD, due to mixed aetiologies	MaND due to AD	MaND due to AD, due to vascular brain disease, due to PD, due to FTLD, due to mixed aetiologies
**Sensitivity**	15–41%	69%	95%	75-83%	78%	79-80%	38%	72-79%	N/A	41%
**Specificity**	87-95%	81%	92%	70-81%	87%	73-78%	99%	80-85%	N/A	N/A

*BNT* Boston naming test, *ANT* Auditory naming test, *VNT* Verbal naming test, *Wo-Fi* Test for finding word retrieval deficits, *ACE-III* Addenbrooke’s cognitive examination III, *MINT* Multilingual naming test, *GBT/GFT/GNT* Graded buildings test/Graded faces test/Graded naming test, *NAB* Neuropsychological assessment battery, *TDQ-30* Test de denomination de Quebec, *MoCA* Montreal cognitive assessment, *MiND* Mild neurocognitive disorder, *MaND* Major neurocognitive disorder, *AD* Alzheimer’s disease, *FTLD* Frontotemporal lobar degeneration, *PD* Parkinson’s disease

#### Stand-alone dysnomia tests

##### Boston naming test (BNT)

The BNT is the most frequently used naming test [44, 45]. Line drawings of objects or animals are presented to the patients who are asked to name them. The tool, which has a satisfactory administration time, has been adapted to different languages (e.g. Danish, Korean, Spanish, Swedish) and has been validated in different cohorts [46–51]. Of note, BNT performance is inversely correlated to age, positively correlated to education and intelligence and is generally higher among cognitively healthy males [6, 52]. BNT has been criticized for including a culturally insensitive item (the noose) [53] as well as for suffering from high false negative rates, inadequate standardization and norms and susceptibility to bias stemming from deficits in visual recognition, visuospatial abilities and/or limited expressive vocabulary of the individual [14, 52, 54–57]. Οf note, the prevalence of vision impairment is approximately 10% in adults in their 70s and exceeds 25% in individuals in their 80s [58]. Moreover, BNT seems to be language specific and perhaps unsuitable for testing bilingual populations [59–61]. Interestingly, the Chinese version of the BNT was recently modified with a set of color pictures to replace the original black-and-white line drawings and the new version was shown to be more adept at identifying amnestic MiND and MaND due to AD [62]. Short versions of BNT have been developed [56, 63]. For instance, the 15- item BNT, which is part of the Consortium to Establish a Registry for Alzheimer’s Disease (CERAD) screening battery for dementia [46, 57, 64, 65], and the 30- item BNT, which comprises all even items from the original 60-item version [66, 67], have been shown to have comparable psychometric properties when contrasted with the original BNT [63].

##### Auditory naming test (ANT)

The ANT is a non-visual test which has a satisfactory administration time, while its scoring lasts approximately 10 minutes. Participants are asked to provide a single word in response to a definition (semantic cue) within 20 seconds. Using this technique visual perception and recognition are bypassed. ANT validity was initially supported by observations that patients with left temporal lobe epilepsy, who manifest naming difficulties, performed worse on auditory but not on visual naming tasks compared to patients with right temporal epilepsy, which is not characterized by dysnomia [68]. ANT has been validated in several different, relatively large studies [6, 45]. The ANT index score enables the simultaneous consideration of correct item response scores, tip-of-the tongue extended latency scores and reaction time per response. Compared to BNT, ANT is particularly sensitive in detecting naming difficulties in patients with MiND, who do not exclusively suffer from memory deficits, in patients with MaND due to AD, cerebrovascular disease or mixed etiologies [6, 45]. No sex related effects on performance on ANT have been unveiled in cognitively healthy older adults, while a positive correlation between education and performance on ANT was detected in healthy individuals aged 50-59 years and 60-69 years [69]. It is noteworthy that the range of ANT scores in cognitively healthy individuals is relatively restricted pointing to ceiling effects [68]. In addition, inconsistent observations have been reported on the utility of ANT to detect age-related decline in auditory naming [45, 69]. Unfortunately, the utility of the ANT has not been studied yet in individuals with ND that display pronounced language impairments, like the language variant of FTLD. Furthermore, performance on ANT is prone to biases pertaining to the common age-related hearing loss [70].

##### Test for finding word retrieval deficits (word Finding = Wo-fi)

The Wo-Fi is a newly formed dysnomia tool, created for the purpose of detecting dysnomia in early stages of AD [24]. It relies of auditory stimuli and has an excellent administration time. According to the findings of the initial, relatively small validation study which included 20 cognitively healthy older adults and 40 patients with MaND due to AD, Wo-Fi has an excellent accuracy. Performance on Wo-Fi is not contingent on either education or sex, but it can be influenced by age- related hearing loss. Replication studies in larger samples, comparing Wo-Fi to other naming tests and assessing Wo-Fi utility in detecting naming capacity impairment in MiND and MaND caused by diseases other than AD (e.g. FTLD, PPA) are urgently needed.

##### Multilingual naming test (MINT)

The MINT is a test which has been designed in such a way that its translation into different languages (e.g., English, Spanish, Mandarin, Hebrew) does not affect item difficulty, since the overall difficulty of MINT items across languages is roughly equivalent [71, 72]. It consists of black-and-white line drawings of objects. Its administration time is satisfactory, as implied by the observation that young healthy individuals succeeded in naming correctly more than 60 items of the MINT sprint, being a new version of the test which includes colored pictures of the objects of the original MINT in addition to a small number of more difficult items drawn from studies designed to elicit tip-of-the-tongue states [73]. Age, education, sex, and race exert significant impact on MINT performance. MINT scores of cognitively normal individuals show a near-ceiling effect being particularly evident in those with higher education (i.e., 13 years and more) [72]. The MINT shows a good accuracy in differentiating between cognitively normal individuals and patients with MaND due to AD [72]. However, it is less effective in distinguishing patients with MiND due to AD from cognitively normal individuals because it contains more easy and medium- difficulty items than is typical for naming tests designed for monolinguals in order to avoid bias stemming from low-proficiency in the non-dominant language [71]. Thus, subtle dysnomia is hardly detected by the MINT. A specially selected 32-item subset of the MINT, containing the items which produce the largest differences between cognitively healthy older adults and patients with AD, successfully captured performance differences between controls and patients in their dominant language, but not in their non-dominant one [71]. A recent study in monolingual Chinese older adults unveiled the necessity of revising the MINT using more universally recognized items of similar word frequency across different cultural and linguistic groups [74].

##### Naming of objects, faces and buildings

Combining the Graded Naming Test (GNT), the Graded Faces Test (GFT) and the Graded Buildings Test (GBT) this instrument has an excellent administration time and is valuable in detecting MiND [43]. In the GNT, participants are asked to name 30 black and white line drawings of objects and animals. The GFT is based on naming the full names of 30 famous faces presented using black and white greyscale images. In GBT participants are confronted with color pictures of 30 famous buildings from across the world (e.g. Buckingham Palace, Taj Mahal). Performance of patients with MiND was found to be lower in all three tests compared to controls, although naming of objects was less affected than naming of faces and buildings [43]. In both groups the pattern of naming accuracy was the same: GNT > GBT > GFT. The results of the discriminant analysis point to the high utility of the combination of the three tests in identifying MiND, justifying the administration of the three tests together. The distribution of GNT scores in healthy individuals was found to be somewhat positively skewed, which coupled with the observed consistent improvement on psychometric test scores over time. This is possibly because improved levels of education may lead to a ceiling effect that limits the GNT’s utility in higher-functioning individuals [75]. Furthermore, performance on naming of objects, faces and buildings is susceptible to both changes over time in the familiarity with test items and to differences between cultural contexts (generational and cultural familiarity, respectively) [75, 76]. More specifically, some items may have become obsolete because of cultural or technological changes, while other items are quite ‘Britain-specific’ (e.g., ‘mitre’, ‘monocle’) [52]. For example, individuals aged 18–29 years systematically under-performed on the GNT items ‘sporran’, ‘periscope’, ‘bellows’, ‘pagoda’, and ‘mitre’ compared to healthy individuals aged 30–79 years [75].

##### Neuropsychological assessment battery (NAB) naming subtest

The naming subtest of the NAB consists of color photographs of common objects, is published as stand-alone measure of naming [52] and has an excellent administration time [77]. NAB naming subtest scores pertain to age and education [78]. Thus, the use of demographically corrected normative adjustments in clinical settings is necessary [77]. In addition, scores are significantly associated with an estimate of premorbid full-scale intelligence. However, the magnitude of the correlations of NAB naming subtest with demographic variables and intelligence is significantly smaller compared to the BNT [79]. A key advantage of the NAB naming subtest is the availability of two alternate forms, minimizing the likelihood of practice effects in cases of serial administration of the subtest. NAB naming subtest demonstrates adequate convergent validity with the BNT. Nevertheless, individuals referred for memory complaints systematically generated higher scores on the NAB than on the BNT [80]. These differences may be attributed to differences in stimuli, cuing procedure, scoring, response time limits, level of item difficulty, and differences in normative sets. The NAB naming test may represent a viable clinical option provided an individual’s performance is interpreted in the context of the individual’s language-, education-, and racial/ethnic background [81]. Nonetheless, independent validation of the NAB naming subtest remains scarce. The distribution of NAB naming subtest values shows a ceiling effect [52] and there are discrepancies between an individual’s performance on the two alternate forms with more individuals obtaining an impaired total score on form 1 compared to form 2, while at the item level, an additional discrepancy is apparent between the two alternate forms for item 30 (‘ostrich’ in form 1; ‘artichoke’ in form 2) [78]. Despite all efforts to access the content of the instrument, the information needed was not provided, since it was property of the publishers.

##### Test de dénomination de Québec (TDQ-30)

The New Color Picture-Naming Test for the Diagnostic of Mild Anomia, TDQ-30, is a relatively demanding instrument with an excellent time of administration [82]. It comprises pictures equally divided between natural and man-made concepts [82, 83]. The distribution of the two types of concepts is equivalent with respect to concept familiarity, imageability, word familiarity, word frequency, word length in syllables and visual complexity. Compared to BNT, TDQ-30 was found to have a higher sensitivity in distinguishing between cognitively healthy older adults and patients with MiND and MaND due to AD. Of note, the sensitivity advantage of the TDQ-30 is mainly attributed to the performance on the natural concepts. The observations of the validation study point out that age and education are significantly related to both total score and the subscore for natural items, while the subscore for man-made items pertains only to age. The findings of the validation study should be treated with caution considering the overrepresentation of highly educated people in the validation study as well as its focus on Caucasian individuals of Quebec. Replication studies in larger clinical samples and assessment of the utility of TDQ-30 in identifying dysnomia in MiND and MaND due to causes other than AD (e.g. FTLD, PPA) are urgently needed.

##### Verbal naming test (VNT)

The VNT is a non-visual test with an excellent administration time [14, 84]. Its development was based on word frequency ratings of spoken language rather than relying on frequency ratings for written language [14]. The items are ordered in decreasing frequency of usage, starting with items of higher frequency (e.g.,“collar”) and ending with more rarely-used words (e.g.,“Nile”). The VNT also includes verbs (e.g.,“melt”) as stimuli and abstract words that would be difficult to depict in pictures (e.g.,“decade”). Performance on the VNT is affected by education and age [14, 84]. The test is susceptible to biases related to hearing deficits, word deafness and auditory agnosia [14] and its utility in identifying dysnomia in MiND or MaND caused by specific neurodegenerative or cerebrovascular diseases, particularly in women, remains largely unknown [24, 84]. Future studies with larger numbers of older men and women of different age groups and educational levels are required.

#### Dysnomia test embedded in global cognitive assessment instruments

##### Naming test embedded in the Addenboorke’s cognitive examination III (ACE-III)

Naming capacity is assessed with a part of the language section of ACE-III [85, 86]. ACE-III naming subtest has an excellent administration time and has been adapted to a plethora of languages (e.g. Chinese, German, Greek, Spanish) [87–90] Of note, performance on former versions of the test battery, such as the ACE-revised, can be converted to ACE III [91–94]. The figure naming section consists of twelve, line drawings of objects or animals which are presented to the examinees who are asked to name them. Based on picture naming properties, such as naming, familiarity, image agreement, and visual complexity, the ACE-III naming test has been modified, within the frames of its adaptation, to different languages and cultural constellations to account for the cultural influences that affect picture naming [93]. A recent report on the psychometric properties of the ACE-III, which relied on an item response theory approach, unveiled that the ACE-III naming subtest has an adequate goodness-of-fit, both to item and model levels, and its items can aid in the discrimination between MaND due to AD and cognitively healthy older adults [95]. Nonetheless, its utility in detecting dysnomia in MiND and MaND caused by other than AD diseases remains unknown. Studies comparing the utility of the ACE-III naming subtest with other established naming tests and assessing the performance of its different versions in identifying both MiND and MaND caused by other the AD diseases are urgently needed, considering the widespread use of the test battery.

##### Naming test embedded in the Montreal cognitive assessment

The MoCA is a commonly used brief screening tool in the detection of both MiND and MaND [96, 97] and is available in different languages (e.g. French, Japanese, Kiswahili, Portuguese, Russian) [98–102]. The MoCA naming subtest includes three line drawings of animals, which differ between the different versions of MoCA, so that cultural differences are taken into account and cultural bias is minimized [103, 104]. Of note, even the stringent cutoff of < 3/3 in the MoCA naming subtest detects only 41% of individuals exhibiting deficits in the language domain [104]. This finding may be attributable to the fact that language deficits in MoCA are captured not only by the naming subtest but also by fluency and sentence repetition tasks. Nonetheless, even combining the scores of all three tasks results in a modest classification accuracy (60%). Studies assessing the validity of different versions of MoCA naming subtest in identifying dysnomia and comparing them to other naming tests are required before final conclusions can be drawn regarding the usefulness of MoCA naming subtest in detecting dysnomia in MIND and MaND.

##### Comparison of test characteristics

**Fig. 3:**
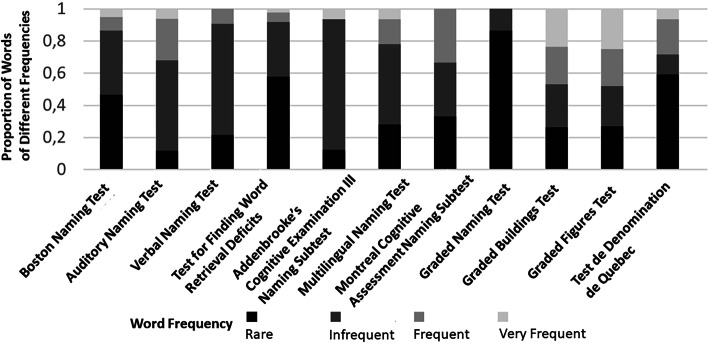
Proportions of words of different frequencies of naming tests that have been validated in neurocognitive disorders

The considered naming tests differ with regard to many dimensions (Table 1). Seven tests are open access while five are available at a cost. The test containing words with the lowest average frequency is the recently published Wo-Fi, while the ANT is the test that includes more commonly used words (Fig. 3). The distribution of test items across the different word frequency categories is most balanced in the case of GBT/GFT. The most extensive assessment is enabled by the BNT which consists of 60 items and its administration lasts 15 minutes. On the other hand, the MoCA naming subtest requires less than 1 min to be administered. Two tests are based on auditory stimuli and ten on visual ones. Most of the latter rely on black and white line drawings, while two rely on color pictures and the GNT/GFT/GBT combine black and white line drawings of objects and animals with black and white greyscale images of famous faces and color pictures of famous buildings. Three tests based on visual stimuli are available in more than one language, while instruments relying on auditory stimuli are exclusively available either in English or in German. All but one test assess naming capacity through correctly named items. Only one test assesses naming capacity based on both naming of nouns and verbs, while all the others rely on naming of nouns. Νine- and nine tests have been validated in MiND and MaND due to AD, respectively, while in the validation studies of five naming tests more than one type of MaND were considered. The sensitivity and specificity of tests in detecting dysnomia in ND vary not only between tests, as expected, but also within tests according to the observations of different validation studies. In one case the variation of sensitivity values exceeds 25% (Table 1).

## Discussion

Since dysnomia is a common aspect of various phenotypes of MiND and MaND, naming tests embody a valuable component of the diagnostic toolboxes of clinicians involved in the diagnostic workup and care of ND. Gaining insights into the characteristics of naming tests may assist clinicians in choosing the most adequate instrument in each setting. Care units differ with regard to aims and principles, needs of service users and staff capacities and needs. Relying exclusively on sensitivity and specificity values in order to make such a decision is a narrow-minded approach. Administration time, stimuli type, test item difficulty and further instrument characteristics affect the compatibility of a tool with both the clinician’s clinical routine and the examinee’s characteristics (e.g. educational level, hearing loss, cognitive impairment severity) [105, 106]. The present review sheds light on such characteristics of dysnomia tests and may facilitate the incorporation of efficient and pragmatic naming tests into clinical practice.

### Naming capacity assessment in primary healthcare services

Primary healthcare increasingly embodies a pivotal player in the diagnosis and care of individuals with ND particularly in the light of their expanding prevalence [107–109]. Relying on the balance between available resources and patient load, primary healthcare staff tries to adjust to the care needs of local communities. Time feasibility of the diagnostic workup of cognitive complaints is vital for both the clinician and the examinee during the diagnostic evaluation process. Moreover, individuals with lower intellectual and/or education level or with severe cognitive impairment are more likely to become frustrated or fatigued during a lengthy and demanding naming assessment [63]. Nonetheless, very short instruments may not detect mild cognitive deficits, which characterize the early stages of ND [5, 6, 8, 10, 11, 14]. For instance, the naming subtest of the MoCA is administered in a few seconds, but it fails to detect dysnomia in more than 50% of individuals with MaND. Despite its longer administration time, more detailed cognitive assessment may facilitate early diagnosis of ND. Of note, in the primary healthcare setting, a thorough cognitive assessment can provide an additional benefit since such tests are more sensitive in unveiling longitudinal cognitive changes. Tracking progression of cognitive impairment is a fundamental issue for disclosing the diagnosis of a ND at the setting of primary healthcare and beyond it [107]. Hence, a reasonable compromise should be reached so that time feasibility is optimally coupled with both reliable assessment and examinee- friendliness of the naming test. The open-access ACE-III naming subtest, consisting of 16 items and lasting less than 5 min, may embody a suitable tool for primary healthcare units. Nonetheless, it is validated only in MaND due to AD. The administration of VNT is longer and includes less frequent items compared to ACE-III naming test, which may lead to examinee’s frustration or fatigue. Nonetheless, VNT has been validated in MiND as well as in MaND caused by different diseases and is open access. Hence, it may also be considered as suitable in primary healthcare settings.

### Naming capacity assessment in secondary- and tertiary brain healthcare services

Compared to primary healthcare settings, assessment of naming ability at memory clinics and secondary and tertiary brain healthcare services is more thorough and not restricted to brief screening tools. An in-depth evaluation of cognitive function is crucial for solving complex diagnostic problems, for detecting even subtle naming deficits and for delineating refractory courses of ND [110]. Naming tests like the TDQ-30 and the NAB naming subtest have an excellent administration time and the TDQ-30 simultaneously enables a detailed evaluation of naming capacity. The TDQ-30 is particularly controlled for psycholinguistic variables, consists of infrequent items and is open-access. It has been validated only in individuals with MiND and MaND due to AD, while the utility of NAB has been evaluated in older adults with MiND and MaND caused by different diseases. If an even more in-depth assessment of naming capacity is required, the BNT includes more infrequent items than TDQ-30, at the expense of a longer administration time. BNT has been validated in patients with MiND and MaND due to different diseases. Although visual perceptual problems are relatively common in aging and prominent in some ND like those caused by Lewy-bodies and may confound the results of visual naming tests, such tests have been historically mainly employed to detect dysnomia [6]. Nonetheless, auditory naming tests like the ANT and the Wo-Fi have recently gained some ground as they bypass biases stemming from visual perceptual deficits and were shown to have a greater validity in detecting very mild word-finding difficulties [6, 45]. A non-visual naming test is useful in distinguishing anomia from visual agnosia, an impairment found in sequelae of stroke and in various neurodegenerative conditions such as posterior cortical atrophy [111, 112]. A possible further advantage of auditory cue naming tests over visual instruments is that auditory naming is more naturalistic since it is more strongly correlated with the context in which word finding impairment is usually expressed in real life i.e. through dialogue and interpersonal conversations rather than as difficulties in naming objects. Wo-Fi consists of less frequent items and its administration time is shorter compared to the ANT. Nonetheless, it has been validated only in individuals with MaND due to AD, while the utility of the ANT has been evaluated in both MiND and MaND caused by different diseases. Additionally, auditory naming assessment with ANT might be less susceptible to bias stemming from limited vocabulary and varying educational levels because its items are more familiar to most examinees possibly at the cost of lower sensitivity in identifying subtle naming deficits [6]. Of note, as auditory impairment is a common comorbidity of ND, the decision between a visual and an auditory extensive naming test should be made on a case-by-case basis.

### Naming capacity assessment and test item lexical frequency

In the present study, special attention has been paid on the frequency, in everyday life, of the words that the individuals are asked to name. Even though the average frequencies of items of all considered naming tests point to at least low frequency, the BNT and the Wo-Fi are the visual and the auditory naming tests, respectively, with the lowest average item frequency. Word frequency is related to the efficiency of word processing [36]. Words of higher frequency pertain to better performance on tasks like word naming, word retrieval, lexical decision (whether a letter string refers to an existing word or not) and semantic decision (whether, for instance, a given word refers to a vegetable or not) [36, 113]. Less frequent words are in general more difficult to name [114]. In several studies word frequency was found to influence naming success of individuals suffering from AD. Interestingly, word frequency was reported, albeit not indisputably, to affect naming capacity disproportionally in AD compared to older adults without ND [115, 116]. Nonetheless, the effect of word frequency on individual naming capacity is not straightforward. It is influenced by factors like the educational level, the assessment of naming capacity in the dominant or in the non-dominant language of the individual or the level of real exposure of the individual to a word [38]. These influences are reflected in the individual differences observed in the effects of word frequency on word processing tasks [117]. Thus, the choice of the most appropriate naming test cannot be founded exclusively on word frequency as a marker of the difficulty of the test. In the process of choosing the most appropriate naming test for a particular clinical setting, the comparison of the average word frequency of the items of different naming tests is a valuable parameter which should be considered together with additional factors like the educational level and the native language of each examinee [117].

### Naming capacity assessment beyond correctly named test items

Naming capacity may only partially be captured through an exclusive focus on correctly named items, as most naming tests do. A more in-depth differentiation of the examinee’s responses to each task may provide valuable information. For instance, in mild MaND due to AD, lexical-semantic errors (including circumlocutions and semantic errors per se) are more common, while such errors seem to diminish as the severity of the syndrome advances and nonresponses (including unrelated responses) increase [118]. From the naming tests considered here, the ANT is the only one that assesses naming capacity not solely based on correctly named items. ANT assessment relies also on tip-of-the tongue extended latency score and on reaction time per response [6, 45]. Taking into account all three variables improves the diagnostic sensitivity of ANT in detecting dysnomia in early MiND. Nonetheless, its administration time, being longer than 15 min, makes it more compatible with hospital-based brain healthcare services and memory clinics than primary healthcare settings.

### Naming capacity assessment and different stimulus types

Different stimulus types seem to differentially affect naming performance. Naming tests are based on different stimulus types with regard to content i.e. natural (plants, vegetables) vs. man-made concepts (vehicles, musical instruments), names of objects vs. faces vs. buildings, nouns vs. verbs, or with regard to the graphical characteristics of the cue (e.g. black and white line-drawn stimuli vs. color photographs). Albeit not indisputable, several studies point to easier naming of man-made concepts than natural concepts both in cognitively healthy older adults and in patients [82, 119]. Hence, natural concepts seem to be more sensitive in unveiling mild naming deficits compared to man-made ones as unraveled in the validation study of TDQ-30 [82, 83]. There are fundamental differences in the structure of semantic knowledge surrounding objects and people. Proper names are more difficultly named, and their trajectory of change is larger compared to common nouns possibly due to the weak and arbitrary links between a proper name and its reference, while the proper name specific retrieval process is intrinsically fragile and source-consuming [120, 121]. In addition, an advantage of naming of buildings over faces has been observed in the validation study of the instrument comprising GNT/GFT/GBT and elsewhere [43, 122]. Nonetheless, identifying and recalling the names of famous people requires a degree of familiarity with them which is contingent on the cultural background and the demographic characteristics of the examinee as well as his/her exposure to the media [120]. Naming of famous buildings like the Taj Mahal also depends on educational level, cultural background and lifestyle. Older adults living in remote areas are often digitally illiterate and may not be familiar with music- and film industry celebrities or famous buildings across the globe and subsequently fail to identify them, even though their naming capacity is not impaired. In such a way the accuracy of the individual’s naming ability assessment is undermined. Moreover, except for ANT all other naming tests considered here rely exclusively on naming of nouns. Naming of verbs is less susceptible to age-related changes compared to the naming of nouns, while aphasic changes can affect differently verb- and noun-naming [123]. Interestingly, the former primarily pertains to frontal cortex activity, while the latter is more related to temporal lobe activity [124]. Furthermore, a recent meta-analysis of 35 studies unveiled that color information had an effect on object recognition and was able to improve naming accuracy and speed correct response times [125]. Color photographs are more ecologically valid and provide a more realistic representation of real-life objects [126]. A recent report points to the higher utility of a color-picture version of BNT compared to the black and white version in differentiating between amnestic MiND and MaND due to AD [62]. Thus, naming tests relying exclusively or partially on color photographs like the TDQ-30 and the GNT/GFT/GBT, respectively, and those including natural concepts may be more efficient in detecting subtle naming deficits compared to instruments including black and white drawings of man-made objects. On the other hand, naming of buildings and famous people is prone to bias stemming from individual low familiarity with famous buildings and/or celebrities while demanding tasks may lead to frustration and premature termination of the assessment. Again, the decision about the most adequate naming test based on the different stimulus types should be made on a case-by-case basis.

### Limitations

The present review has several limitations. Neither psychometric properties of naming tests like content validity, internal consistency, construct validity, nor the linguistic characteristics of their items (e.g. word length, similarity to other words, contextual diversity, imageability) are presented here, since such properties have been thought to be beyond the scope of a narrative review aiming to support healthcare professionals seeking tests compatible with their routine clinical endeavors to detect dysnomia in people with ND. Moreover, NAB was not considered in the item frequency comparison analysis, since its content is property of its publishers. In addition, despite the importance of the word frequency effects on naming capacity, there are further factors crucially affecting it [36], that are not taken into account here. Furthermore, only open-access and available for purchase instruments validated in detecting dysnomia in ND are considered here, which leaves various naming tests out of the pool.

## Conclusions

To conclude, twelve open-access or available for purchase naming tests have been validated in ND. They differ with regard to their features. Primary healthcare services would benefit most from naming tests embodying a reasonable compromise between short administration time and high accuracy in detecting even mild naming deficits and their changes over time, like the ACE-III naming subtest and the VNT. On the other hand, at secondary and tertiary brain healthcare services visual or auditory extensive naming tests like TDQ-30, NAB and BNT or ANT and Wo-Fi, respectively, enable an in-depth evaluation of naming function. Nonetheless, large validation studies and the cultural adaptation of tests currently available in very limited languages to additional languages are urgently needed, so that the wide employment of such instruments in everyday clinical endeavors across the globe is catalyzed.

## Supplementary Information

**Additional file 1: Table 1S.** Dysnomia instruments screened for fulfilling the study eligibility criteria.

## Data Availability

The datasets used and analyzed during the current study are available from the corresponding author on reasonable request.

## Abbreviations

ACE-III: Addenbrooke’s cognitive examination III
AD: Alzheimer’s disease
ANT: Auditory naming test
BNT: Boston naming test
CERAD: Consortium to Establish a Registry for Alzheimer’s Disease
CIND: Cognitive impairment no dementia
FTLD: Frontotemporal lobar degeneration
GBT/GFT/GNT: Graded buildings test/Graded figures test/Graded naming test
MaND: major neurocognitive disorder
MiND: mild neurocognitive disorder
MINT: Multilingual naming test
MoCA: Montreal cognitive assessment
NAB: Neuropsychological assessment battery
ND: Neurocognitive disorder
PPA: Primary progressive aphasia
TDQ-30: Test de denomination de Quebec
VCI: Vascular cognitive impairment
VNT: Verbal naming test
Wo-Fi: Test for finding word retrieval deficits
