# Supplementary material for: Naming ability assessment in neurocognitive disorders: a clinician’s perspective
Source: BMC Psychiatry. 2022 Dec 30;22:837. doi: 10.1186/s12888-022-04486-x (PMC9801565; doi:10.1186/s12888-022-04486-x)
Supplement: Supplementary file 1 — Additional file 1: Table 1S. Dysnomia instruments screened for fulfilling the study eligibility criteria. [file 12888_2022_4486_MOESM1_ESM.docx]

**Naming ability assessment in neurocognitive disorders: A clinician’s perspective**

Eliza (Eleni-Zacharoula) Georgiou, Department of Psychiatry, Patras University General Hospital, Faculty of Medicine, School of Health Sciences, University of Patras, Patras, Greece, elizageo8@gmail.com

Savvina Prapiadou, Department of Psychiatry, Faculty of Medicine, School of Health Sciences, University of Patras, Patras, Greece, sprapiadou@gmail.com

Vasileios Thomopoulos, Large-Scale Machine Learning & Cloud Data Engineering Laboratory (ML@Cloud-Lab), Faculty of Computer Engineering & Informatics, School of Engineering, University of Patras, Patras, Greece, vthomopoulos@upatras.gr

Maria Skondra, Psychogeriatric unit for neurocognitive assessment and caregiver counseling, Patras Office of The Hellenic Red Cross, Patras, Greece; Department of Psychiatry, Faculty of Medicine, School of Health Sciences, University of Patras, Patras, Greece, mskon@hotmail.gr

Marina Charalampopoulou, Department of Psychiatry, Patras University General Hospital, Faculty of Medicine, School of Health Sciences, University of Patras, Patras, Greece, marina.charalamp@gmail.com

Asimina Pachi, Department of Psychiatry, Faculty of Medicine, School of Health Sciences, University of Patras, Patras, Greece, asiminapachi@gmail.com

Αlexandra Anagnostopoulou, Department of Psychiatry, Faculty of Medicine, School of Health Sciences, University of Patras, Patras, Greece; General Hospital of Zakynthos “Saint Dionysios”, Zakynthos, Greece, [anagnostopouloualex@gmail.com](mailto:anagnostopouloualex@gmail.com)

Theofanis Vorvolakos, Department of Psychiatry, Faculty of Medicine, School of Health Sciences, University Hospital of Alexandroupolis, Democritus University of Thrace, Alexandroupolis, Greece, tvorvola@med.duth.gr

Robert Perneczky, Division of Mental Health in Older Adults and Alzheimer Therapy and Research Center, Department of Psychiatry and Psychotherapy, University Hospital, Ludwig-Maximilians-Universität Munich, Munich, Germany; Ageing Epidemiology (AGE) Research Unit, School of Public Health, Faculty of Medicine, The Imperial College of Science, Technology and Medicine, London, UK; German Center for Neurodegenerative Diseases (DZNE) Munich, Munich, Germany; Munich Cluster for Systems Neurology (SyNergy), Munich, Germany; Sheffield Institute for Translational Neurosciences (SITraN), University of Sheffield, Sheffield, UK, Robert.Perneczky@med.uni-muenchen.de

Antonios Politis, First Department of Psychiatry, Eginition Hospital, School of Medicine, National and Kapodistrian University of Athens, Athens, Greece; Department of Psychiatry, Division of Geriatric Psychiatry and Neuropsychiatry, Johns Hopkins Medical School, Baltimore, USA, apolitis@med.uoa.gr

Panagiotis Alexopoulos, Department of Psychiatry, Patras University General Hospital, Faculty of Medicine, School of Health Sciences, University of Patras, Patras, Greece; Global Brain Health Institute, Medical School, Trinity College Dublin, The University of Dublin, Dublin, Republic of Ireland; Department of Psychiatry and Psychotherapy, Klinikum rechts der Isar, Faculty of Medicine, Technical University of Munich, Munich, Germany; Patras Dementia Day Care Center, Corporation for Succor and Care of Elderly and Disabled – FRODIZO, Patras, Greece, panos.alexopoulos@upatras.gr

**Table 1S** Dysnomia instruments screened for fulfilling the study eligibility criteria

| **TEST** | **KEYWORDS**  Dysnomia, anomia, naming and major neurocognitive disorder, mild neurocognitive disorder, dementia, mild cognitive impairment, cognitive impairment no dementia, Alzheimer’s disease, vascular cognitive impairment, frontotemporal lobar degeneration, frontotemporal dementia, primary progressive aphasia, semantic dementia | **ANOMIA/ DYSNOMIA detection capacity assessment** | **Tested in impaired population** | **Open Access or Purchase Option** | **Inclusion?** |
| --- | --- | --- | --- | --- | --- |
| **Naming and Oral Reading for Language in Aphasia 6-point scale (NORLA-6)** | No | - | - | - | No |
| **Philadelphia Naming Test** | No | - | - | - | No |
| **Sentence Production Test (SPT)** | No | - | - | - | No |
| **Cologne Picture Naming Test (CoNaT)** | No | - | - | - | No |
| **Categorical Naming Test (CNT)** | No | - | - | - | No |
| **Srole Scale** | No | - | - | - | No |
| **The apraxia of speech rating scale** | No | - | - | - | No |
| **Pictures naming Cuetos** | No | - | - | - | No |
| **Snodgrass- Vanderwart** | No | - | - | - | No |
| **The Alzheimer’s Disease Assessment Scale–Cognitive Subscale (ADAS-Cog)** | Yes | No | - | - | No |
| **The British Sign Language Cognitive Screening Test** | Yes | No | - | - | No |
| **Pyramid and Palm Trees Test (PPTT)** | Yes | No | - | - | No |
| **Birmingham Object Recognition Battery (BORB)** | Yes | No | - | - | No |
| **Western Aphasia Battery (WAB)** | Yes | No | - | - | No |
| **Cambridge Semantic Memory Test Battery (CSB)** | Yes | No | - | - | No |
| **Verbal fluency (FAS)** | Yes | No | - | - | No |
| **Peabody Picture Vocabulary Test – (PPVT)** | Yes | No | - | - | No |
| **Boston Diagnostic Aphasia Examination (BDAE)** | Yes | No | - | - | No |
| **Test for the Reception of Grammar (TROG-2)** | Yes | No | - | - | No |
| **The northwestern anagram test** | Yes | No | - | - | No |
| **Kissing and dancing** | Yes | No | - | - | No |
| **Progressive Aphasia Severity Scale PASS** | Yes | No | - | - | No |
| **SEntence Comprehension Test (SECT) and Make A Sentence Test (MAST)** | Yes | No | - | - | No |
| **Short form of the Pyramids and Palm Trees Test** | Yes | No | - | - | No |
| **The repeat and**  **point test** | Yes | No | - | - | No |
| **The northwestern naming battery** | Yes | No | - | - | No |
| **The Nombela naming test** | Yes | No | - | - | No |
| **Repeatable Battery for the Assessment of Neuropsychological Status (RBANS)** | Yes | No | - | - | No |
| **Wechsler Memory Scale (WMS)** | Yes | No | - | - | No |
| **Progressive Aphasia Language Scale (PALS)** | Yes | No | - | - | No |
| **Sydney language**  **Battery (SYDBAT)** | Yes | No | - | - | No |
| **Cross-linguistic naming test, Ardila** | Yes | Yes | No \| norms | - | No |
| **Psycholinguistic Assessments of Language Processing in Aphasia (PALPA)** | Yes | Yes | No | - | No |
| **Memory for Names Test (Mem4Names)** | Yes | Yes | Yes | No | No |
| **Graded Buildings Test** | Yes | Yes | Yes | Yes | Yes |
| **Graded Naming Test** | Yes | Yes | Yes | Yes | Yes |
| **Graded Faces Test** | Yes | Yes | Yes | Yes | Yes |
| **Neuropsychological Assessment Battery (NAB) Naming Test** | Yes | Yes | Yes | Yes | Yes |
| **Auditory Naming Test (ANT)** | Yes | Yes | Yes | Yes | Yes |
| **Addenboorke’s cognitive examination III language section (ACE-III)** | Yes | Yes | Yes | Yes | Yes |
| **Verbal Naming Test (VNT)** | Yes | Yes | Yes | Yes | Yes |
| **Boston Naming Test (BNT)** | Yes | Yes | Yes | Yes | Yes |
| **Test for finding word retrieval deficits** | Yes | Yes | Yes | Yes | Yes |
| **Test de dénomination de Québec (TDQ-30)** | Yes | Yes | Yes | Yes | Yes |
| **Multilingual Naming Test 32-items (MINT)** | Yes | Yes | Yes | Yes | Yes |
| **Montreal Cognitive Assessment Naming Subtest** | Yes | Yes | Yes | Yes | Yes |
